# The impact of changes to heroin supply on blood-borne virus notifications and injecting related harms in New South Wales, Australia

**DOI:** 10.1186/1471-2458-5-84

**Published:** 2005-08-16

**Authors:** Carolyn Day, Louisa Degenhardt, Stuart Gilmour, Wayne Hall

**Affiliations:** 1 National Centre in HIV Epidemiology and Clinical Research, University of New South Wales Level 2, 376 Victoria Street, Darlinghurst, NSW 2010, Australia; 2 National Drug and Alcohol Research Centre University of New South Wales, Sydney, NSW 2052, Australia; 3 Office of Public Policy and Ethics Institute for Molecular Bioscience, University of Queensland, St Lucia, Brisbane, 4072, Australia

## Abstract

**Background:**

In early 2001 Australia experienced a sudden and unexpected disruption to heroin availability, know as the 'heroin shortage'. This 'shortage has been linked to a decrease in needle and syringe output and therefore possibly a reduction in injecting drug use. We aimed to examine changes, if any, in blood-borne viral infections and presentations for injecting related problems related to injecting drug use following the reduction heroin availability in Australia, in the context of widespread harm reduction measures.

**Methods:**

Time series analysis of State level databases on HIV, hepatitis B, hepatitis C notifications and hospital and emergency department data. Examination of changes in HIV, hepatitis B, hepatitis C notifications and hospital and emergency department admissions for injection-related problems following the onset of the heroin shortage; non-parametric curve-fitting of number of hepatitis C notifications among those aged 15–19 years.

**Results:**

There were no changes observed in hospital visits for injection-related problems. There was no change related to the onset heroin shortage in the number of hepatitis C notifications among persons aged 15–19 years, but HCV notifications have subsequently decreased in this group. No change occurred in HIV and hepatitis B notifications.

**Conclusion:**

A marked reduction in heroin supply resulted in no increase in injection-related harm at the community level. However, a delayed decrease in HCV notifications among young people may be related. These changes occurred in a setting with widespread, publicly funded harm reduction initiatives.

## Background

Injecting drug use is an important risk factor for the transmission of blood-borne viral infections (BBVI) such as the human immunodeficiency virus (HIV) and the hepatitis C virus (HCV) [1]. Harm reduction strategies such as needle and syringe programs have been instrumental in maintaining low HIV prevalence among injecting drug users (IDU) in settings where they were implemented early [2], such as Australia [3,4]. Reducing the prevalence and incidence of HCV, however, has proved more challenging with continuing high prevalence among injecting drug users in Australia [5,6] and elsewhere [7,8] and high incidence among young injectors [9]. IDU are also subject to increased risk of other injection-related problems such as abscesses and thromboses [10,11].

Caulkins has suggested that illicit drug-related harms are influenced by drug price [12]. A significant relationship was found between drug prices, and mentions of cocaine and heroin in United States Emergency Departments, whereby admissions increased as drug prices decreased [12]. These findings raise the question: Would injecting-related harms including BBVI *decrease *if drug prices *increased *and availability decreased [13]? The answer to this question, until now, has been based upon theory rather than evidence, because sharp reductions in drug supply have rarely occurred. In this paper, we take advantage of recent changes in the Australian heroin market to answer this question empirically.

In early 2001, there were consistent reports of a dramatic decline in the availability of heroin in New South Wales (NSW) [14,15], where previously heroin had been readily available at historically low price and high purity [16]. Australia's Illicit Drug Reporting System revealed a reduction in heroin supply across Australia, reflected by reduced purity and availability, and a marked increase in price [17]. This 'heroin shortage' was most severe from January to April 2001 [18]. The market began to stabilise after this date but heroin availability has not returned to pre-shortage levels, and the heroin market in Australia appears to have been altered in structure [18].

The reduced heroin supply resulted in at least some former primary heroin users substituting a range of other drug classes for heroin during the shortage, in particular cocaine [15,19,20]. Changes in the heroin market was also associated with a significant decrease in the distribution of needles and syringes, a proxy measure for injecting drug use [21].

This change is important because increased prevalence of cocaine injection has been associated with a number of adverse consequences. The HIV outbreak during the mid 1990s in Vancouver has been attributed in part to increased cocaine injection [22]. It has since been found to be an independent predictor of HIV infection [23] and related to high levels of Emergency Department use for drug related problems, most notably soft tissue infection [24]. Given that heroin use has driven the hepatitis C epidemic in Australia, it is unclear what impact changes in patterns of drug use will have on the epidemic [25].

An increase in benzodiazepine injection was also reported to occur at the time of the shortage [19]. Benzodiazepine injection is associated with a range of harms, including soft tissue damage and increased BBVI risk [26,27]. Harms such as these were reported in qualitative accounts of the shortage by both IDU and key informants.

These changes in drug availability and patterns of injecting drug use occurred in a setting in which harm reduction strategies were widely used. Publicly funded NSPs were introduced to Australia in 1987 [28], and methadone maintenance programs which were established in the 1970s were significantly expanded in 1985 and again in 1999 [29]. More recently, a Medically Supervised Injecting Centre was trialled in one of the key NSW drug markets [30]. Although needle and syringe sharing continues to be reported in Australia, the available evidence suggests that the prevalence of this behaviour has remained stable at 15–20% sharing in the past month [31].

Given these changes in drug use in an established harm reduction setting, this study aimed to examine population level changes in harms associated with injecting drug use, such as HCV, HIV and hepatitis B (HBV) that occurred after the change in heroin supply in NSW, the State containing Australia's largest heroin markets [32]. Specifically, the study examined:

1. Changes, if any, in the number of notifications of BBVI cases including the hepatitis B virus (HBV), HCV and HIV. In particular trends in HCV notifications among young persons, who are at greatest risk of infection [9,33] and among whom prevalence provides a reasonable approximation of incidence were examined; and

2. Changes, if any, in ED and hospital data on presentations for injection-related problems (possibly as a result of the increased use of other drugs such as cocaine and benzodiazepines).

## Methods

### Data

New cases of HCV, HBV and HIV must be notified by either doctor or laboratory to the State Health Department where they are recorded in the Notifiable Diseases Database and the HIV/AIDS database. Notification of these infections is mandatory in Australia, thus all laboratories are legally obligated to notify all positive results. Notification includes date of birth and detailed name codes, thereby reducing duplicate notifications. De-identified data were retrieved from these databases on the number of individuals diagnosed by age and gender. Monthly data by date of diagnosis on diagnoses were available from 1997.

Although not all HBV and HIV notifications are due to injecting drug use (indeed in Australia, very few HIV infections are likely to be [3]), this is true across all time points examined, and there is no extraneous reason to believe that this pattern would have changed over the period examined. In the case of HCV, more than 90% of such cases occur through injecting drug use [34], thus any changes in these data are very probably directly related to injecting drug use.

Due to the often asymptomatic nature of HCV, not all new diagnoses represent acute or newly acquired infections. Given this, notifications among young IDU are considered those most likely to reflect new hepatitis C infections, or incident cases. Large proportions of IDU (>60%) report recent HCV testing [31]. Thus data on HCV notification among young people was analysed separately.

Diagnostic information on persons presenting to the NSW hospital EDs is recorded at the time of presentation and is coded using the International Classification of Diseases, 9^th ^Revision (ICD_9). The NSW Emergency Department Collection is a database of information collected from approximately a third of NSW EDs. Because these are mostly the larger EDs in NSW, the Collection represents approximately two thirds of all NSW emergency patients. The following codes were used to examine injection-related problems: unspecified septicaemia (038.9); acute and subacute endocarditis (421.0); other peripheral vascular disease (443.9); phlebitis and thrombophlebitis (451.0); other venous embolism and thrombosis (458.8); cellulitis and abscess (681.00, 681.10, 682.0–9); other local infections of skin and subcutaneous tissue (686.9) and chronic ulcer of skin (707.1, 707.9).

### Data analysis

Ideally time series of the sort presented here would be analysed using intervention time series models, which allow estimation of the effect of posited interventions in the series after adjusting for the relationships which exist between different values over time in many time series data (serial dependence). For HCV notifications amongst 15–19 year olds, the intervention model time series was not appropriate because the series showed different behaviour in different time periods, so a loess smoother [35] was fitted to this data series using S-Plus 6.1. Such a smoother fits a line to the data using a series of linear regressions fitted in a small neighbourhood of points. For this data the size of this neighbourhood (the span) was estimated using Generalised Cross Validation [36]. Such methods enable the shape of the modelled data to be estimated based only on the data, and avoid subjective decisions about details of the plot, such as the point at which periods of different behaviour occur (changes in slope) or the precise point of onset of sudden changes in level (steps) [36]. This enabled the researchers to avoid making, for example, subjective judgements about exactly when HCV notifications began to decline. Other time series had small counts and no formal time series analysis was conducted – these series were inspected for signs of large-scale changes at the point of the heroin shortage.

## Results

### Blood borne viral infections (BBVIs)

**Figure 1:**
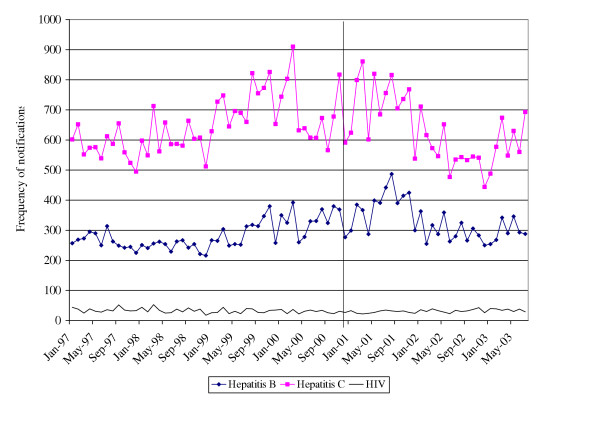
**Number of HIV, Hepatitis B and C notifications, NSW 1997 – 2003**. NDD and HIV/AIDS databases, Communicable Diseases Branch, NSW Health Department.

**Figure 2:**
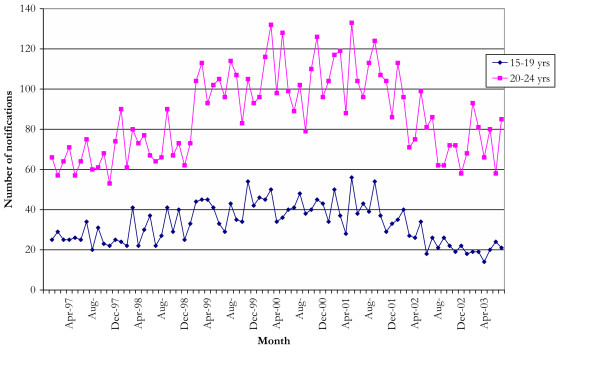
**Hepatitis C notifications among 15–19 and 20–24 year olds, NSW 1997–2003**. NDD and HIV/AIDS databases, Communicable Diseases Branch, NSW Health Department.

**Figure 3:**
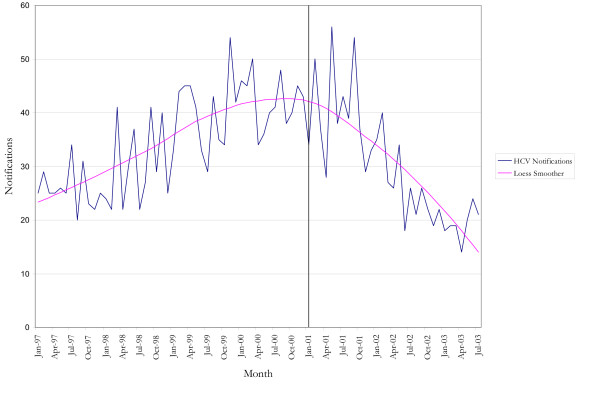
**observed and predicted numbers of Hepatitis C Virus notifications in 15–19 year olds, 1997 – 2003**. NDD and HIV/AIDS databases, Communicable Diseases Branch, NSW Health Department.

The possibility of changes in BBVIs was examined using data on NSW notifications of HBV, HCV and HIV infection. There were no apparent differences in the overall number of total notifications following the onset of the shortage for HIV, HCV or HBV (Figure 1). However, the number of HCV and HBV notifications for persons aged 15–19 years, the groups most likely to be effected, were examined (Figure 2). Cross-correlation functions indicated that there was no significant relationship between those functions which describe the heroin shortage and the HCV notifications data at plausible positive lags. A loess smoother (span = 0.75) was fitted to the HCV notification data in order to describe the general structure of the series (Figure 3), and indicated that the series peaked several months before the onset of the heroin shortage, with a downward trend in the series after this point.

There is no evidence that this downward trend changed at the time of the heroin shortage or that there were transient non-random increases in the series after the shortage. Unfortunately the series was not amenable to ARIMA methods and more informative analysis could not be conducted in the absence of plausible evidence from cross-correlation functions of a shortage effect on the series (as described above).

### Injection-related problems

**Figure 4:**
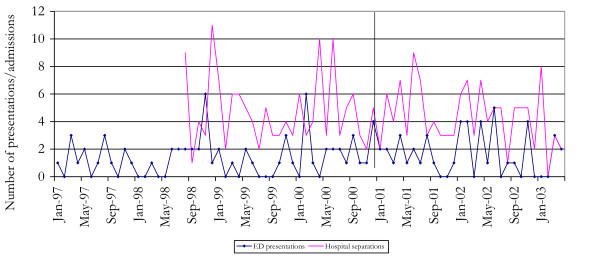
**Number of drug related ED admissions and hospital separations for injection-related problems, NSW 1997 – 2003**. Emergency Department Information System, NSW Department of Health; and NSW Department of Health.

Figure 4 shows the number of ED admissions and hospital separations where injection-related problems were noted, from 1997–2003. No formal time series was conducted due to the small numbers of such admissions noted each month. No noticeable increase occurred following the onset of the shortage.

## Discussion

This study found no increase in notifications for HIV, HCV or HBV following the reduction in heroin supply. Given that only a proportion of HIV and HBV cases are attributable to injecting drug use, this is perhaps not surprising HIV and HBV. However, the clear reduction in HCV cases among those aged 15–19 years, commencing slightly before the onset of the reduction in heroin supply, was consistent with declines in other indicators of heroin-related harm in the community, including a State-wide 28% reduction in needle and syringe distribution [21]; a 63% decrease in ambulance callouts, a 40% decrease in emergency department admissions for non-fatal heroin overdose and a 43% decrease in heroin related deaths [37]; and a 45% decrease in heroin possession/use offences [38]. This suggests that the decrease in HCV notifications in this group is probably related to the extent of heroin injection in the community. There are no alternative explanations for the decrease in notifications, which was not predicted by mathematical models of the hepatitis C epidemic in Australia [39].

The most plausible explanation of the reduction in HCV notifications among young people is that fewer young persons were initiating injecting drug use. This suggests that there has been a reduction in the extent of injecting drug use and hence probably fewer persons, particularly younger persons, at risk of contracting BBVIs [9,40]. If this is the case, any reductions in HCV notification related to the heroin shortage may be subject to a lag, given the period between initiation to injecting and HCV seroconversion. The extent of this lag is unclear, but USA data suggest that many new HCV infections occur within months of initiating injecting [41]. The proportion of HCV positive IDUs injecting for less than three years has varied between 13% and 22% [5,25]. However, given the uncertainly of such a lag, it could not be validly modelled as any intervention term included, other than the date of the heroin shortage, would have been chosen arbitrarily.

Moreover, evidence from the loess smoother showed that the HCV notifications data series among young people had levelled off before the shortage. Evidence from this same smoother showed that it began to decline some time after the shortage. This behaviour of the data series is possibly explained by the shortage.

Despite reports of increased injecting risk, no increase in other BBVI was reported. Any increase in BBVI risk attributable to the shortage would most likely have occurred during the peak period of the shortage, a relatively brief period of approximately four months [18]. The rate of HIV infection among IDU in Australia is probably too low to produce dramatic increases in the number of HIV infections in that time, even if the risk of infection was increased [42]. Moreover both HIV and HBV are more easily and, in Australia, more commonly sexually transmitted and would therefore be less responsive to any changes in injecting drug use.

It is important to note that these changes occurred in a setting in which harm reduction measures were readily available to IDU. It is unclear whether similar trends would be observed in countries where the prevalence of HIV among IDU is higher, and where access to harm reduction measures is more limited. Estimates suggest that IDU in Australia would need to share needles/syringes with 31 others over a year to increase the prevalence of HIV among IDU in Australia [42]. In contexts where new injecting equipment is not as readily available as in Australia, or where the use of cocaine is much more widespread, changes in heroin availability may have a different effect.

There was also no evidence of increased injection-related harm, as measured by changes to the number of presentations at hospitals for injecting-related problems. This was despite concern about injection-related problems related to frenetic or risky injecting of benzodiazepines and cocaine by some IDU [43,44]. It seems most likely that either these changes occurred among a relatively small group, and/or that the resulting problems were relatively minor and treated by general practitioners or other primary health care services. The number of these infections attributable to injecting drug use is also likely to be small. However, a Canadian study found IDU have a high level of emergency department utilisation, predominantly for injection related problems, especially soft-tissue infection, and this is most common among cocaine users and frequent injectors [24].

## Limitations

This study has relied on secondary data sources as indirect measures of the complications of injecting drug use. As changes at the *population *level were examined, notifications were the most appropriate data. As notification of HIV, HBV and HCV is mandatory in Australia and cases are typically reported by the testing laboratory, these data are unlikely to be subject to the reporting bias reported by others [e.g. [45]]. In Australia, more than 90% of HCV infections are related to injecting drug use [31], and infections among 15–19 year olds are most likely to be newly acquired. Nonetheless, the clear decrease in HCV infection among young people is not easily explained by any other hypotheses, and is consistent with other research on the consequences of the heroin shortage [46].

Several of the conclusions presented here are based on visual inspection of data series with small numbers, rather than on statistical analysis. The authors do not believe that observable effects in these series would be considered of public health importance even if statistical significance could be shown, and consider visual inspection suffices in these instances.

## Conclusion

This research has found that following a reduction in heroin supply there was no change in HIV or HBV at the population level, but there was an apparent decrease in HCV infections among those aged 15–19 years. These changes occurred in a setting with widespread, publicly funded harm reduction initiatives.

## Abbreviations

ARIMA Auto Regressive Integrated Moving Average

BBVI Blood-borne viral infections

ED Emergency department

HCV Hepatitis C virus

HBV Hepatitis B virus

HIV Human immunodeficiency virus

IDU Injecting drug users

NSP Needle and syringe program

NSW New South Wales

## Competing interests

The author(s) declare that they have no competing interests.

## Authors' contributions

All authors contributed to the paper. C.Day, L.Degenhardt and W.Hall conceived the study. L.Degehnardt supervised the research. S.Gilmour led the analysis and C.Day led the writing. All authors helped to conceptualise ideas, interpret the findings and reviewed drafts of the manuscript.

## Pre-publication history

The pre-publication history for this paper can be accessed here:


